# RETRACTED: Bartlett et al. Uremic Toxins Activates Na/K-ATPase Oxidant Amplification Loop Causing Phenotypic Changes in Adipocytes in In Vitro Models. *Int. J. Mol. Sci.* 2018, *19*, 2685

**DOI:** 10.3390/ijms262110605

**Published:** 2025-10-31

**Authors:** David E. Bartlett, Richard B. Miller, Scott Thiesfeldt, Hari Vishal Lakhani, Tilak Khanal, Rebecca D. Pratt, Cameron L. Cottrill, Rebecca L. Klug, Nathaniel Seth Adkins, Paul C. Bown, D. Blaine Nease, Joseph I. Shapiro, Komal Sodhi

**Affiliations:** 1 Department of Internal Medicine, Marshall University Joan C Edwards School of Medicine, Huntington, WV 25755, USA; bartlett14@marshall.edu (D.E.B.); miller1088@marshall.edu (R.B.M.); thiesfeldt@marshall.edu (S.T.); lakhani@marshall.edu (H.V.L.); khanal@marshall.edu (T.K.); martin570@marshall.edu (R.D.P.); cottrill41@marshall.edu (C.L.C.);; 2 Department of Surgery and Biomedical Sciences, Marshall University Joan C Edwards School of Medicine, Huntington, WV 25701, USA; klug3@marshall.edu (R.L.K.); nsa18@yahoo.com (N.S.A.); bown@marshall.edu (P.C.B.); blaine.nease@yahoo.com (D.B.N.)

The journal retracts the article titled “Uremic Toxins Activates Na/K-ATPase Oxidant Amplification Loop Causing Phenotypic Changes in Adipocytes in In Vitro Models” [1], cited above.

Following publication, concerns were brought to the attention of the Editorial Office regarding image duplication within this article [1].

Adhering to our standard procedure, the Editorial Office and Editorial Board conducted an investigation that confirmed a duplication within the Western blot bands presented in Figures 2C and 4C. Given the impact of this issue on the validity of the results, the Editorial Board and the authors have determined that the overall findings could not be relied upon and decided to retract this publication [1], as per MDPI’s retraction policy (https://www.mdpi.com/ethics#_bookmark30 (accessed on 1 January 2020)).

This retraction was approved by the Editor-in-Chief of the journal *IJMS*.

The authors disagreed with this retraction.
